# Effect of maternal education and encouragement on newborn care utilization: a health system intervention

**DOI:** 10.1186/s12887-021-02773-2

**Published:** 2021-07-21

**Authors:** Ali-Asghar Kolahi, Mohsen Abbasi-Kangevari, Alireza Abadi

**Affiliations:** Social Determinants of Health Research Center, Shahid Beheshti University of Medical Sciences, Koodakyar Ave., Daneshju Blvd., Velenjak, Tehran, Iran

**Keywords:** Infant care, Infant health, Neonatal screening, Neonatal care

## Abstract

**Background:**

The objective of this health system interventional study was to determine the effect of delivering newborn-care-oriented education and encouragement on newborn care utilization.

**Methods:**

This study was performed in the urban health centers of the catchment area of Tehran Defined Population, which covered 10 of the 22 municipality districts of Tehran. The two catchment areas included 10,000 families in the intervention and 20,000 families in the control areas. As many as 4837 newborns (interventio*n* = 1544, control = 3293) were enrolled and followed until the end of the second month of life. The utilization of the three newborn care visits, as recommended by national guidelines, was compared among the intervention and control groups.

**Results:**

As many as 877 (56.8%) newborns in the intervention group and 1214 (36.9%) in the control group received all their three newborn care visits. The mean number of newborn care visits was higher in the intervention group compared to the control group: 2.26 (0.99) versus 1.84 (1.07), *p* < 0.001. The number of newborns who did not attend any of their three newborn care visits was 143 (9.3%) in the intervention group and 468 (14.2%) in the control group.

**Conclusions:**

The intervention improved newborn care utilization during the first 2 months after birth. It could be suggested that active follow-up be added to newborn care guidelines. Parents need to be informed of the necessity and benefits of newborn care and be encouraged to perform all three newborn care visits.

## Background

Newborn care (NBC) and screening are essential in preventing, early diagnosis of treatable disorders, managing health conditions, and reducing neonatal mortality. About three million infants die every year in the first month of life, primarily due to preventable causes, including pneumonia, preterm birth complications, neonatal intrapartum-related complications, diarrhea, neonatal sepsis, and malaria [1]. Therefore, the implementation of Countdown to 2015 for Maternal, Newborn, and Child Survival has resulted in a reduction in worldwide neonatal mortality from 26 a year to 19. The Countdown to 2015 initiative monitored the coverage of priority interventions to achieve the Millennium Development Goals to reduce maternal and child mortality [2, 3]. Moreover, Sustainable Development Goal 3 has set 12 neonatal deaths or less per 1000 live births by 2030 [2, 4].

NBC’s quality depends on the initiation and continuation of NBC, components of care, and care provider. Although the postnatal period is a critical phase in newborns’ lives, and most neonatal deaths occur during this time, the quality of care has received improper attention [5]. In addition, newborn screening implementation differs among different countries, especially in the Middle East and North Africa, where there is diversity in population size, income, health systems, and insurance coverage [6].

The schedule of NBC in Iran was changed in 2005. Before that, the newborn immunization started with Bacillus Calmette–Guérin (BCG), poliomyelitis, and hepatitis B at the time of birth in the hospital. The continuation of immunization took place 45 days after birth for diphtheria, pertussis, and tetanus (DPT), poliomyelitis, and a second dose of hepatitis B at Urban Health Centers (UHC) [7]. Therefore, most newborns would be brought to UHC for the first time on day 45 after birth, and the first newborn care visit (NBCV) was scheduled for that time.

In 2005, the immunization time for DPT, poliomyelitis, and hepatitis B was changed from day 45 to day 60 after birth. In addition, screening tests in the 3–5 days after birth were added [8]. Since the World Health Organization recommends at least three NBCVs in the first 2 months after birth [9], three newborn care visits were added to NBC’s schedule. However, our field observations indicated that the three NBCVs were not sufficiently performed in UHCs or private sectors. We assumed that parents had insufficient knowledge about NBC, and therefore do not demand it. Field observations also suggested that health workers showed resistance against providing NBC services unless demanded by parents. It needs to be mentioned that UHCs are public health facilities with free-of-charge services. The private sector includes pediatricians’ offices.

The objective of this study was to determine the effect of education and encouragement to receive newborn care on newborn care utilization in the first 2 months after birth by conducting an intervention with a concurrent control group.

## Methods

This study was approved by the Ethical Committee of Shahid Beheshti University of Medical Sciences under the reference code IR.SBMU.RETECH.REC.1396.848 and the study methodology conformed to Helsinki Declaration standards as revised in 1989. All data remained confidential, and participants provided informed consent before taking part in the study. Participants provided informed consent before taking part in the study. In addition, participation was entirely voluntary. Participants could opt out of the study at any time, and the decision to leave the study would not affect the conventional quality of newborn care services.

### Overview

This health system intervention was performed in the urban health centers of the two catchment areas of Tehran Defined Population, which covered 10 of the 22 municipality districts of Tehran. The two catchment areas included 10,000 families in the intervention and 20,000 families in the control areas. The census method was used for the selection of both intervention and control groups. The intervention group included all 1544 newborns in intervention areas of TDP who were born from November 2017 until August 2018 and were referred to UHCs for mandatory newborn screening tests. The study’s control group included all 3293 newborns born in the same period in control areas of TDP and were referred to UHCs for newborn screening tests. Newborns were followed until the end of the second month of life. The intervention included newborn-care-oriented education and encouraging parents to utilize NBC services. The control group received conventional care at UHCs. The utilization of newborn care was compared among the intervention and control groups.

### Design

This study was performed in the two catchment areas of Tehran Defined Population (TDP). TDP includes two areas, intervention and control, which form a defined population representative of Tehran [10, 11].

**Fig. 1:**
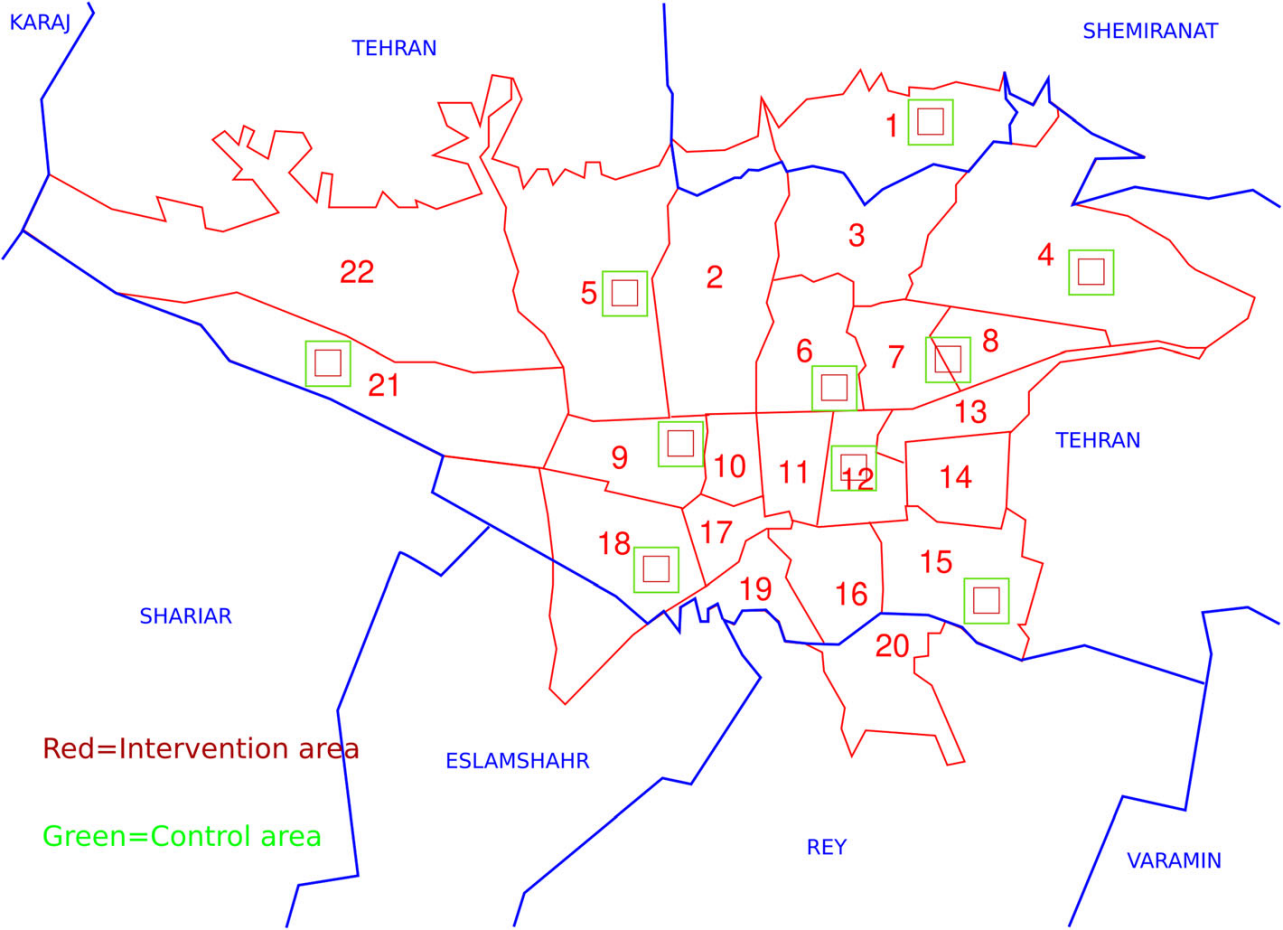
Location of the intervention and control areas on the map of Tehran

The intervention areas include 10,000 families, 40,000 individuals under the coverage of health services provided by 10 UHCs in 10 municipality districts of the total 22 districts in Tehran. These ten districts consist of two districts from each of the five socioeconomic regions of the city, including north, east, center, west, and south, according to the Tehran Urban Health Equity Assessment and Response Tool study (Urban HEART) [12]. To care for potential selection bias, the control areas are were selected to be the neighboring areas of the intervention areas, as presented in Fig. 1. The control areas include almost 20,000 families, 80,000 individuals. Thus, the intervention group and the control group would be matched in terms of socioeconomic status and access to healthcare.

### Newborn care organization in Iran

Both public and private sector provide newborn care services in Iran:

- The public sector

Like all other Iran cities, NBC and screening tests in Tehran are available in the public sector for all newborns in UHCs free of charge. All newborns need to complete at least 17 NBCVs, which start from the third day after birth to 72 months. This study focuses on the first three NBCVs, which have recently been added to NBC’s schedule, which are to be performed in the first 2 months after birth. According to the national guideline, the first visit should be performed in the first 3–5 days of life, the second in 14–15 days, and the third in 30–45 days. The guideline of Iran is consistent with the World Health Organization recommendations on postnatal care of mother and newborn [13, 14]. The main components of NBC include assessment of the general appearance of the newborn, neonatal jaundice, weight, height, head circumference, vision, and developmental milestones; immunization; iron and folic acid supplementation; and counseling about breastfeeding, nutrition, maternal and neonatal hygiene, and family planning [14]. Screening tests are mandatory and should be carried out three to 5 days after birth. The tests include screening for phenylketonuria, congenital hypothyroidism, and glucose-6-phosphate-dehydrogenase deficiency. Screening tests of newborns who require hospitalization are carried out in the hospitals [8]. Midwifery graduates provide NBC in the UHCs. Newborns are referred to the attending general practitioners in the UHCs in cases of warning signs such as low birth weight, jaundice, infection, poor milk sucking, cyanosis, craniosynostosis, and not reaching the developmental milestones. The general practitioners then refer newborns to pediatricians if needed [14]. It is noticeable that the first dose of oral poliomyelitis, hepatitis B virus; and BCG vaccines were administered for neonates at hospital at the time of discharge.

b. The private sector

Newborns could receive NBC services from the private sector at the pediatricians’ office and the hospitals’ pediatric clinics, for which they will be charged. Laboratories of hospitals or private laboratories also provide screening tests. The out-of-pocket costs vary based on the type of medical insurance.

Unlike the public sector, the focus of the pediatricians in the private sector is more on treatment rather than prevention. Although they usually follow the national guidelines of NBC in terms of the schedule and the treatment measures, they do not emphasize prevention aspects, supporting breastfeeding, and maternal education as much as healthcare providers in the public sector. The reason could be the lack of enough training on prevention in the medical curriculum [15].

### Intervention

The intervention included newborn-care-oriented education and encouraging parents to utilize NBC services. This process encompassed providing in-person health education, reinforcing the vital role of prompt NBC in the wellbeing of newborns, explaining the components of a standard NBCV, and distributing healthcare-oriented pamphlets and booklets. Healthcare providers in UHCs were assigned verbal education of parents in the intervention group about the role of NBC in improving their newborns’ health, based on a pre-prepared passage, which was the same in all the UHCs. They would also encourage and remind parents to bring their children for newborn care. Mothers in the control group would receive the conventional NBC services with the same quality from midwives in a shared room in the UHC. Less time would be allocated for their education or encouragement.

### Inclusion criteria

The inclusion criteria were living in the catchment area for the intervention group and the surrounding neighborhoods for the control group and parents’ consent to participate. Newborns who had severe health conditions and therefore were admitted to hospital or needed follow-up by specialists, died in the first 2 months of life or were lost track of due to reasons like moving out of the covered districts of TDP were excluded.

### Participants

The census method was used for the selection of both intervention and control groups. The intervention group included all newborns in intervention areas of TDP who were born from November 2017 until August 2018 and were referred to UHCs for mandatory newborn screening tests. The study’s control group included all newborns born in the same period in control areas of TDP and were referred to UHCs for newborn screening tests.

**Fig. 2:**
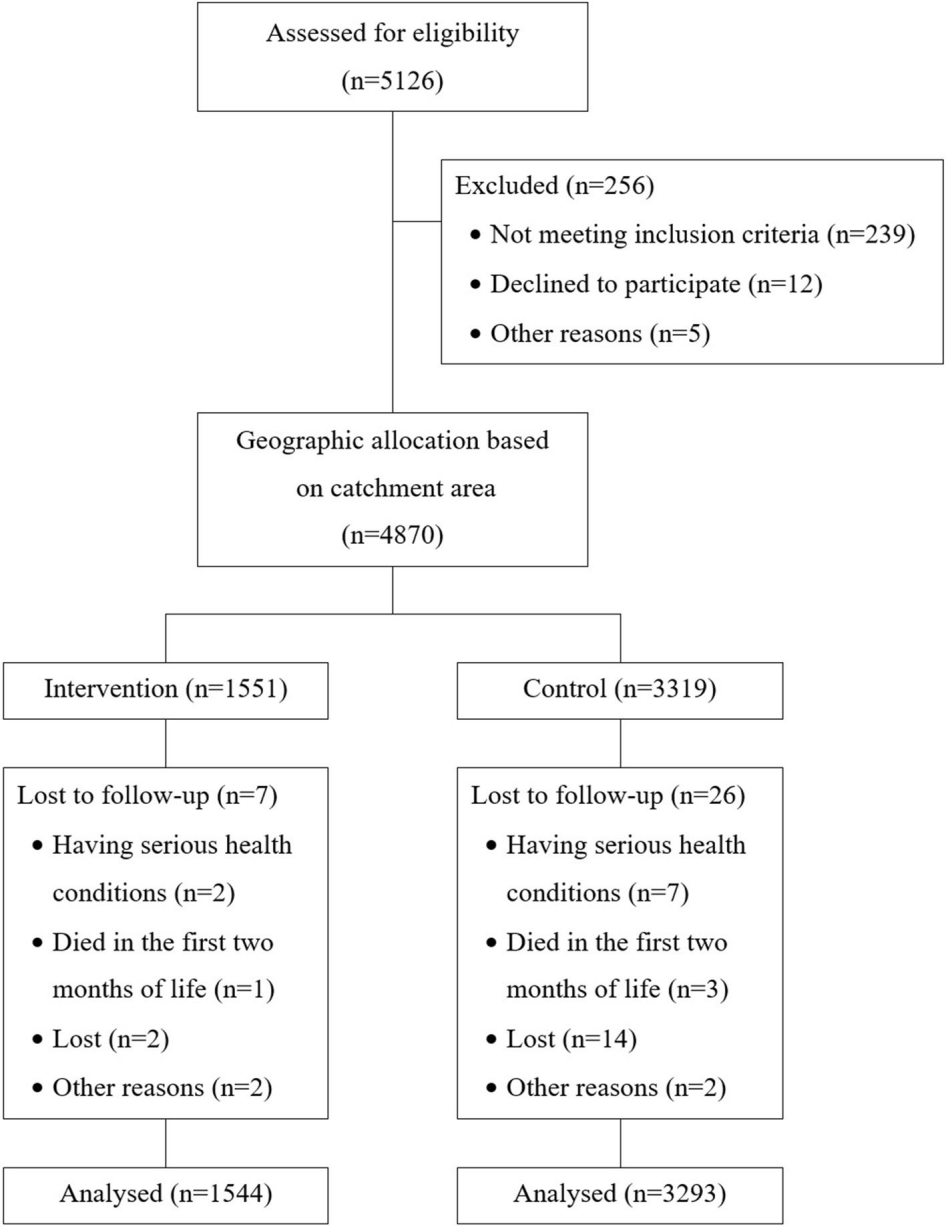
Flowchart of sampling

Among 4870 (intervention = 1551, control = 3319) newborns included in the study, 33 were excluded from, and finally data of 4837 (intervention = 1544, control = 3293) were analyzed. A more detailed flowchart for sampling is shown in Fig. 2.

### Variables and data collection

Variables included parents’ sociodemographic characteristics, mothers’ age at the time of marriage, number of alive children, type of birth, place of screening tests, time and place of NBCVs, healthcare providers, and whether families were encouraged to perform NBC while going for the screening tests. Data were recorded at the time of visits or collected from written medical records.

### Data analysis

Frequency, mean, and standard deviation were used to describe the data. We used the chi-Square test for categorized variables. T-test and one-way analysis of variance (ANOVA) test were used to analyze the differences among means of two groups and three groups or more, respectively. Multivariable modeling was used to control for confounding variables. Variables significant at a *p* < 0.2 in a univariate model were included in the multivariate model, and no confounders were found among the independent study variables. Statistical analyses were performed using IBM SPSS Statistics 21. A probability level of less than 0.05 was considered significant.

## Results

### Sociodemographic characteristics

The mean (SD) age of mothers was 29.3 (4.9) in the intervention group and 29.9 (5.0) in the control group. The mean (SD) age of mothers at the time of marriage was 22.5 (4.3) in the intervention group and 22.8 (4.6) in the control group. The mean (SD) age of fathers was 33.7 (5.4) in the intervention group and 34.3 (5.8) in the control group. Other sociodemographic characteristics of parents are presented in Table 1.

**Table 1:** Sociodemographic characteristics of parents in both groups

Demographic variable	Frequency (%)	
Characteristics of mothers	Intervention *n* = 1544	Control *n* = 3293
Literacy		
Illiterate	14 (0.9)	18 (0.5)
Primary school	63 (4.1)	189 (5.7)
Middle school	178 (11.5)	216 (6.6)
High school and University	1289 (83.5)	2870 (87.1)
Occupation		
Employed	233 (15.1)	531 (16.1)
Unemployed	1311 (84.9)	2762 (83.9)
Birth method		
Normal	321 (20.8)	702 (21.3)
Caesarian section	1223 (79.2)	2591 (78.7)
Number of alive children		
One	865 (56.0)	1817 (55.2)
Two	566 (36.7)	1152 (35.0)
Three of more	113 (7.3)	324 (9.8)
Medical insurance		
Social Security	1024 (66.3)	2091 (63.5)
Medical Services	157 (10.2)	326 (9.9)
Other	176 (11.4)	405 (12.3)
None	187 (12.1)	471 (14.3)
Characteristics of fathers		
Literacy		
Illiterate	10 (0.6)	27 (0.8)
Primary school	95 (6.2)	153 (4.6)
Middle school	273 (17.7)	431 (13.1)
High school and University	1166 (75.5)	2682 (81.5)
Occupation		
Professional	85 (5.5)	178 (5.4)
Technician	122 (7.9)	385 (11.7)
Clerical	349 (22.6)	764 (23.2)
Services	295 (19.1)	612 (18.6)
Craft worker	356 (23.1)	375 (11.4)
Machine operator	111 (7.2)	204 (6.2)
Elementary worker	140 (9.1)	369 (11.2)
Armed forces	50 (3.2)	324 (9.8)
Others	36 (2.3)	82 (2.5)

### Newborn care utilization

Some 57% of newborns in the intervention group and 37% in the control group received all their three NBCVs. The number of newborns who did not complete all their three NBCVs and had one or two NBCVs was higher in the control group. In addition, 9% of newborns in the intervention group and 14% in the control group did not receive any NBCVs. Newborn care utilization among the intervention and control group is presented in Table 2.

**Table 2:** Newborn care utilization and healthcare facilities for screening tests among intervention and control group

Variable	Intervention		Control		*P*-value
Number of NBCVs	n (%)	95% CI	n (%)	95% CI	
All three	877 (56.8)	54.3, 59.3	1214 (36.9)	35.2, 38.5	< 0.001
Two	332 (21.5)	19.4, 23.6	819 (24.8)	23.4, 26.3	< 0.001
One	192 (12.4)	10.8, 14.1	792 (24.1)	22.6, 25.5	< 0.001
None	143 (9.3)	7.8, 10.7	468 (14.2)	13.0, 15.4	< 0.001

The number of newborns who received NBCVs increased from the first to the third visit, as 1007 (65.2) newborns in the intervention group, and 1583 (48.1) in the control group received their first NBCV; 1176 (76.2) newborns in the intervention group and 1880 (57.1) in the control group received their second NBCV, and 1304 (84.5) newborns in the intervention group and 2609 (79.2) in the control group received their third NBCV. The number of NBCVs was higher among newborns in the intervention group, those brought to UHCs for screening tests, and those whose parents were encouraged to perform NBCVs. In addition, attendance in the first NBCV was higher among newborns born via normal vaginal delivery (Table 3). No significant correlations were found between the mean number of NBCVs and number of alive children, mothers’ age, their level of education, the age of mothers at the time of marriage, type of birth, and fathers’ age, level of education, their job, and whether their family has medical insurance.

**Table 3:** Determinants of newborn care utilization among various groups

Determinants		Number of NBCVs		*P*-value
		Mean (SD)	95% CI	
Study group	Intervention	2.26 (0.99)	2.21–2.31	
	Control	1.84 (1.07)	1.81–1.88	< 0.001
Healthcare facility for screening tests	UHCs	2.02 (1.06)	1.98–2.05	
	Other facilities	1.78 (1.06)	1.71–1.86	< 0.001
Being encouraged to perform NBCVs	Yes	2.47 (0.83)	2.44–2.51	
	No	1.45 (1.04)	1.41–1.50	< 0.001
		Attendance in the first NBCV		
		n (%)	95% CI	
Birth type	Normal vaginal delivery	524 (51.2%)	48.1–54.3	
	Caesarian section	1474 (38.6%)	37.1–40.2	< 0.001

### Healthcare facility for newborn care

Among all newborns, 721 (51.5) in the intervention group and 953 (33.7) in the control group received all their three NBCVs in UHCs; 28 (1.8) in the intervention group, and 108 (3.3) newborns in the control group at pediatricians’ office; and 23 (1.5) in the intervention group, and 18 (0.5) newborns in the control group at pediatric clinics of hospitals. Other newborns received NBC services from more than one healthcare provider. Therefore, the proportion of each healthcare provider was calculated according to the number of total NBCVs. Of the total 4632 NBCVs for the intervention group, three per newborn, 3487 (75.3) were received. Of the total 9879 NBCVs for the control group, three per newborn, 6072 (61.5) were received. UHCs were the main healthcare facilities for NBC, as 3046 (87.4) visits in the intervention group and 5064 (83.4) in the control group were performed in UHCs.

### Healthcare facility for screening tests

The main place for screening tests was UHCs. Other healthcare facilities for screening tests are presented in Table 4. Only six newborns in the intervention group and 18 newborns in the control group did not have their screening tests. As many as 1188 (76.9) parents in the intervention group and 1286 (39.1) parents in the control group had been encouraged to perform NBCVs while going for screening tests. In addition, newborns who did their screening tests in UHCs were more likely to complete all the three NBCVs compared to those who did their screening tests in pediatricians’ office or pediatric clinic in hospital: 1826 (45.6%); 95% CI [44.0, 47.1] versus 265 (32.0%); 95% CI [28.8, 35.1], *p* < 0.001.

**Table 4:** Healthcare facilities for screening tests among the intervention and the control group

Facility	Intervention n (%)	Control n (%)
UHCs	1345 (87.5)	2663 (81.3)
Hospitals	159 (10.3)	513 (15.7)
Laboratories	18 (1.2)	72 (2.2)
Other	16 (1.0)	27 (0.8)

### The accompanying person for the first newborn care visit

As many as 2590 newborns received the first NBCV: 546 (21.0) newborns were born through normal birth, among whom 492 (90.1) newborns were brought to healthcare facilities by their mothers for the first NBC; and 2046 (79.0) newborns were born through caesarian section, among whom 1553 (75.9) were brought to healthcare facilities by their mothers.

## Discussion

The study showed that 57% of newborns in the intervention group received all their three NBCVs during the first 2 months after birth, which is 54% higher than the control group. This indicates that the intervention improved NBC utilization. Similarly, some studies reported that active follow-up of newborns, especially during the first days of life, could increase the likelihood of NBCV being duly performed [16–18]. The study interventions’ effect is beyond completing the newborn care in the first 2 months of life, as mothers who feel the newborn care is a perceived need will probably pursue the following visits with more responsibility.

The more attendance rate of newborns in the intervention group could suggest that healthcare providers’ health education and recommendations have been successful in making NBC become a perceived health need among parents. The current need for education among parents could be considered a golden opportunity for healthcare systems to deliver accurate information and training. Some 90% of new fathers in a study in China expressed their need for further education on infant screening tests and immunization [19]. Choosing effective measures and proper means of education is particularly significant among younger couples, who may encounter negative feelings or anxiety as new parents undergo the role transition period. A systematic review showed that providing protocol-based health information and advice on appropriate care-seeking, health practices, and reminders via text messaging or using hotlines could increase newborn care attendance and vaccination rate [20].

Newborns’ attendance at NBCVs increased from the first to the third visit. Newborns born through normal birth received the first NBCV more than those born through caesarian section, which could be justified because 90% of newborns born through normal vaginal delivery were brought to healthcare facilities by their mothers for their screening tests and NBC. In contrast, 76% of whom were born via caesarian section were brought by their mothers. The birth type has been associated with other healthcare services after birth, as well. A study reported that mothers who gave birth to their children via caesarian section were less likely to attend postpartum care visits [21].

Notably, 79% of all newborns in the study were born via caesarian section, highlighting the impact of birth type on performing NBC. The prevalence of the caesarian section was similar to our study on prenatal care utilization in Tehran [10]. Mothers who had caesarian sections would be at rest during the early days of the postpartum period. Therefore, some newborns would be brought by someone other than their mothers and get hungry, irritable, and miss their mothers during their visit to the health facilities and therefore miss their NBCV. Therefore, for mothers who cannot make it to NBCVs, it could be suggested that NBC services be provided at home. Some studies have suggested that delivering NBC at home is more effective and an ideal alternative for traditional office-care [22–25].

Almost all newborns, 99.6%, did their screening tests. After the birth, parents were told in the hospital that their newborns needed to take the screening tests. Similarly, the coverage rate of newborn screening tests in Turkey was 99%, although newborns get screened for biotinidase deficiency instead of G6PD deficiency [26]. Although the successful implementation of national newborn screening faces multiple challenges, proper communication and encouragement are considered to be among the effective means of attracting parental support [27].

UHCs were the leading facilities for both newborn care and screening tests. Parents who did the screening tests of their newborns in UHCs were more likely to be encouraged to receive NBC services. In addition, newborns who did their screening tests in UHCs were more likely to complete all the three NBCVs. The reason could be that other newborns had gone to healthcare facilities for screening tests such as laboratories, where NBC could not be provided.

### Strengths and limitations

This is the first population-based interventional study to focus on the effect of encouragement and active follow-up on bringing newborns for NBCVs and utilizing newborn care services in Tehran. The study was conducted in the Tehran Defined Population catchment area, which forms a defined population representative of Tehran. The study included an intervention group and a control group, who were selected via census sampling from the same catchment area to eliminate the confounding variables. Both groups were matched in terms of socioeconomic status. In addition, the study included a large sample and multiple UHCs.

Newborns who, due to any reason, needed to be hospitalized or receive further care by specialists could not be followed, which was the limitation of the study.

## Conclusion

The intervention improved newborn care utilization during the first 2 months after birth. We suggest that active follow-up be added to the guidelines of newborn care. Parents need to be informed of the necessity and benefits of newborn care, and be encouraged to perform all three newborn care visits.

## Data Availability

The data that support the findings of this study are available from Social Determinants of Health Research Center, Shahid Beheshti University of Medical Sciences, Tehran, Iran. However, restrictions apply to the availability of these data, which were used under license for the current study, and so are not publicly available. Data are however available from the authors upon reasonable request and with permission of Shahid Beheshti University of Medical Sciences.

## Abbreviations

NBC: Newborn care
DPT: Diphtheria, pertussis, and tetanus
UHC: Urban Health Centers
NBCV: Newborn care visit
TDP: Tehran Defined Population
